# Unsupervised Early Detection of Physical Activity Behaviour Changes from Wearable Accelerometer Data

**DOI:** 10.3390/s22218255

**Published:** 2022-10-28

**Authors:** Claudio Diaz, Corinne Caillaud, Kalina Yacef

**Affiliations:** 1 School of Computer Science, The University of Sydney, Sydney, NSW 2006, Australia; 2 Biomedical Informatics and Digital Health, School of Medical Sciences, Faculty of Medicine and Health, The University of Sydney, Sydney, NSW 2006, Australia; 3 Charles Perkins Centre, The University of Sydney, Sydney, NSW 2006, Australia

**Keywords:** behaviour changes, physical activity, unsupervised learning, activity tracker, accelerometer

## Abstract

Wearable accelerometers record physical activity with high resolution, potentially capturing the rich details of behaviour changes and habits. Detecting these changes as they emerge is valuable information for any strategy that promotes physical activity and teaches healthy behaviours or habits. Indeed, this offers the opportunity to provide timely feedback and to tailor programmes to each participant’s needs, thus helping to promote the adherence to and the effectiveness of the intervention. This article presents and illustrates U-BEHAVED, an unsupervised algorithm that periodically scans step data streamed from activity trackers to detect physical activity behaviour changes to assess whether they may become habitual patterns. Using rolling time windows, current behaviours are compared with recent previous ones, identifying any significant change. If sustained over time, these new behaviours are classified as potentially new habits. We validated this detection algorithm using a physical activity tracker step dataset (N = 12,798) from 79 users. The algorithm detected 80% of behaviour changes of at least 400 steps within the same hour in users with low variability in physical activity, and of 1600 steps in those with high variability. Based on a threshold cadence of approximately 100 steps per minute for standard walking pace, this number of steps would suggest approximately 4 and 16 min of physical activity at moderate-to-vigorous intensity, respectively. The detection rate for new habits was 80% with a minimum threshold of 500 or 1600 steps within the same hour in users with low or high variability, respectively.

## 1. Introduction

Wearable accelerometry-based activity sensors are widely used to objectively, continuously, and unobtrusively record and monitor the physical activity (PA) of subjects in free-living conditions and produce a quantitative assessment of PA [1]. They provide detailed PA data that may hold valuable information on PA behaviours and habits. By retrieving activity sensor data regularly, behaviour changes can be detected as they emerge, helping to identify new behaviours in real time and to determine whether they are repeated over time, suggesting new PA habits. Detailed real-time information may be crucial to provide support and feedback, or to understand PA behaviour changes in the context of interventions or programmes promoting PA or teaching healthy behaviour habits. These can be critical in the fight against overweight and obesity because PA contributes to weight control. Increasing PA is considered a progression toward healthy behaviours.

The early detection of recent changes in PA behaviour enables timely encouragement and re-enforcement of the newest healthy behaviours while discouraging unhealthy behaviours. The real-time automated detection of behaviour changes and/or new habits is needed to trigger timely personalised feedback that can allow self-reflection, the adjustment of PA goals to each participant, and the real-time individualisation of the contents and pace of a health intervention programme.

Many of the existing methods for detecting PA behaviour changes have been designed to analyse historical data, focusing on the detection of significant changes that occurred between specific moments (e.g., pre- and post-intervention). Others use specific PA thresholds to flag a pre-defined achievement (e.g., when the number of daily steps becomes higher than 10K). However, these methods cannot detect subtle but significant behaviour changes in real time. Indeed, behaviour changes can occur in small increments over a certain period.

This article describes the development of Unsupervised BEhaviour and HAbit Very Early Detector (U-BEHAVED), a novel unsupervised machine learning technique that detects significant day-to-day changes in PA behaviour using step data from wearable sensors. The research questions were:

RQ1. Can PA behaviour changes be periodically detected using step data from activity trackers?
RQ2. Can we detect whether these PA behaviour changes are sustained over time (suggesting a new habit)?

The article is structured as follows. Section 2 presents previous work on the detection of PA behaviours by sensor data mining. Section 3 discusses the challenges of detecting PA behaviour changes. Section 4 describes and illustrates the U-BEHAVED algorithm. Section 5 reports the U-BEHAVED algorithm’s accuracy in detecting PA changes from sensor data. Section 6 concludes the article.

## 2. Related Work

A diverse number of machine learning techniques use data from wearable sensors to detect PA behaviour changes. The techniques can be grouped into supervised, unsupervised, and semi-supervised methods to identify changes during PA promotion interventions (real time) or after the interventions (not real time).

### 2.1. Supervised Machine Learning Techniques

Supervised machine learning techniques have been used to classify sensor-derived PA data by training a model with pre-classified sensor-derived PA data. In educational scenarios, researchers rely on this approach to identify the improvement or correction of physical movements towards expert standards, for example, specific body movements in martial arts [2] and dance [3] or physical interactions with clinical equipment [4]. However, these techniques are not suitable to detect PA behaviour changes when the objective is to compare current PA behaviours with past ones. As PA behaviours differ between individuals, a specific volume of PA increases may represent a behaviour change for one individual but not for another. For instance, for a sedentary person, walking 10 extra minutes in the morning can be a significant PA increase, thus signalling a PA behaviour change. Conversely, for a very active person, walking 10 extra minutes may be insignificant compared with their normal volume of physical activity, and may not be considered a PA behaviour change. Therefore, the classification of behaviour changes must be adapted for each person and is unique at each point in time because behaviours constantly evolve. These features do not allow for training a supervised model.

### 2.2. Unsupervised Machine Learning Techniques

Unsupervised machine learning techniques have been used to create sets of similar PA behaviours that are analysed to identify PA behaviour changes. We review them under two sub-groups: (i) those aiming at analysing pre- and post-intervention data and (ii) those for real-time detection.

(i) A first group of studies analysed health education behaviour changes after an intervention (post-intervention analysis, not in real time). For instance, Ref. [5] analysed the impact of an intervention using a k-means algorithm to group the time spent at different PA levels (PAL) and step goal achievements. Ref. [6] assessed PA patterns during an intervention using k-means clustering to group the participants’ hourly steps. Ref. [7] evaluated the impact of an intervention by grouping the participants’ PA bouts using a k-means algorithm. Ref. [8] analysed PA patterns in women throughout the day using k-means clustering to group their daily metabolic equivalent (MET) per minute. Ref. [9] assessed the impact of sharing personal PA behaviours in an online community using agents. Lastly, Ref. [10] developed a window-based algorithm to detect and analyse changes in participants’ behaviours after an intervention using time series of participants’ steps captured by wearable sensors. All these studies detected changes in PA behaviours; however, they focused on PA behaviour changes after the intervention (post-intervention analysis) and used the participants’ behaviours during the whole intervention to determine significant behaviour changes. These techniques are not suitable for our purpose because they work once all the PA data recording period is available, and they aim to identify the most prominent behaviour changes among all behaviours over the recording period. In contrast, we aim to detect significant behaviour changes as they appear. Such an early and timely detection of behavioural changes is key to provide quick support and feedback for promoting physical activity.

(ii) A second group of unsupervised techniques analyse the participants’ PA behaviour changes during interventions to promote PA (real-time analysis). For instance, Ref. [11] helped participants to examine their daily behaviour by grouping their PA using a k-means algorithm to cluster the mean heartbeat and oxygen saturation values. Ref. [12] generated daily personalised text messages with custom timing, frequency, and feedback about their step count/goal and with motivational content to support reflection using a multiarmed bandit (MAB) algorithm and the number of minutes spent in PA. Ref. [13] used a genetic algorithm, pareto-optimality, and the participants’ daily sleep duration, steps, calories, exercise duration, exercise distance, exercise calories, step count, step distance, and step calories to analyse the wearer’s data and make personal lifestyle improvement recommendations. Ref. [14] recommended PA to participants using an agglomerative cluster technique that grouped MET values, heart rate, gender, height, weight, age, and exercise time, type, and frequency. Ref. [15] personalised the intervention for each participant using an MAB to model the participant’s days in the intervention, PA goal compliance, weight, food intake, and calories. Ref. [16] explained the PAL dynamics in a community using a social contagion model to model the steps and results of a psychological questionnaire on self-efficacy, barriers, social norms, long-term goals, intentions, satisfaction, outcome expectations, and models. Ref. [17] generated personalised suggestions to help users reach their PA behaviour goals using MAB and PA frequency and calories. Ref. [18] adapted the step goal settings of the intervention for each participant using a behavioural analytics algorithm, the daily steps, and a goal. Ref. [19] used an MAB for step modelling, in addition to motivation and psychometric data to personalise the social comparison among participants with the aim of motivating them towards increasing their PA behaviour. Finally, Ref. [20] developed a reinforcement learning recommendation system that used clustered daily segments of participants’ steps and sleep behaviours to provide personalised suggestions of PA patterns to achieve weight-loss effects. This second group of unsupervised techniques used a threshold to detect when a PA change is important: a step goal, a cluster (sorted by PA), or any other measure.

### 2.3. Semi-Supervised Machine Learning Techniques

Some studies used thresholds to train classification algorithms, creating semi-supervised techniques to detect PA behaviour changes. For instance, Ref. [21] provided personalised daily activity recommendations using shallow neural networks to process PA and demographics, attitudes, intentions, and habit data from questionnaires. Ref. [22] created gamified personalised feedback using a random forest technique and a weighted score to model the PA change from accelerometry data. Ref. [23] delivered personalised feedback to participants about their progress to help them achieve their personal step goal using a random forest technique to model the hour when PA work was performed, the hourly number of steps for that hour, the number of steps made in the past hour, the cumulative number of steps up to that hour, and the mean number of steps on workdays. Lastly, Ref. [24] personalised feedback time and content using k-nearest-neighbour and support vector machine techniques to model PA physical variables (not specified).

### 2.4. Summary of Related Work

Although unsupervised and semi-supervised algorithms detect real-time individual behaviour changes in PA-promotion contexts, they rely on two different methods: comparing the aggregated PA data or determining whether a predefined PA objective is met. When using aggregated PA data, all details of any significant behaviour change below the aggregated level are diluted and cannot be detected. For example, at the daily level, it would be possible to detect a significant PA increase in a given day, but without details on whether, when, and how many different behaviour changes occurred during that day. Similarly, when using a PA objective, any PA change below that goal is not detected. For instance, if a sedentary participant adds a new PA during a day (e.g., going for a walk) but does not reach the step goal, this behaviour change is not detected. Conversely, our objective is to detect all significant behaviour changes, including small and subtle behaviour changes, because future habits are progressively built on past habits, generating notable behaviour changes.

In conclusion, there are successful algorithms to detect behaviour changes. Conversely, methods to identify real-time progressive behaviour changes in the framework of PA promotion interventions and healthy behaviour teaching are lacking. Here, we extended these previous works on detecting PA behaviour changes by creating an unsupervised machine learning technique that identifies the participants’ PA behaviour changes hourly. We also propose a method to recognize sustained PA behaviour changes that suggest new habits.

## 3. Challenges of Detecting Physical Activity Behaviour Changes in Health Education

PA behaviour changes are indicated by differences in the step number between days; however, not all step differences are true behaviour changes. Indeed, step differences between days can be observed due to the natural variability of the usual daily PA [25,26], making the step difference magnitude to be considered an undefined behaviour change.

PA variations are highly individual. Therefore, the *first challenge* is to identify significant step differences while taking into account the variations of each participant.

Even people who maintain regular daily PA are expected to show some variability in their execution time and step number. For instance, a person who runs every morning will not run exactly at the same time and perform exactly the same number of steps. The *second challenge* is to avoid flagging habitual PA with shifted execution time and similar step number as a behaviour change.

When a PA behaviour change is detected, it could either be a transient variation not sustained in time or the beginning of a new behaviour. Therefore, the *third challenge* is to flag PA behaviour changes that are maintained over time, suggesting that they are potentially becoming habits.

To exploit the patterns detected in a health education programme, it is important that they are detected in a timely manner, which means shortly after they occur. The *fourth challenge* is processing large amounts of PA data almost in real time to detect PA behaviour changes briefly after they occur.

## 4. Methods

The U-BEHAVED algorithm uses continuous and streaming PA data from activity trackers. Data are pre-processed, resulting in a time series of hourly steps coarse enough to detect intra-day changes and avoid mislabelling any energy burst (e.g., a short sprint) as a behaviour change. To detect the behaviour changes as they occur, the algorithm first computes the usual behaviour by building a rolling time window that creates a time series of the mean number of steps per hour. Next, to measure the magnitude of the behaviour change, the algorithm calculates the difference between the hourly steps of each day and the mean number of steps per hour. Outliers of the hourly step difference are classified as behaviour changes using a second rolling time window to avoid incorrectly flagging habitual PA with negligible changes in execution time and step number as changes. Finally, to flag a continuous behaviour change as a new habit, the algorithm moves the outlier limits from the day when the initial behaviour change is detected to the subsequent days.

The algorithm extends the work discussed in Section 2 and relies on time series anomaly detection of residuals [27] and interquartile ranges (IQR) [28]. In the next section, we explain in detail the data requirements to serve as input to U-BEHAVED, how data must be pre-processed, the algorithm steps, and the resulting outputs.

### 4.1. Dataset Requirements

The input dataset must be a discrete time series of steps, regularly spaced at the input sampling rate, and synchronised with U-BEHAVED at regular intervals. We used an hourly interval as a sensible time interval to identify changes in human activity. However, other regular intervals can also be used. Datasets from any PA tracker device that can be transformed into number of steps with ≤1 h sampling can be used, such as step data from (or computed from) commercial wearable devices (e.g., Fitbit), smartphone devices, and research-grade devices (e.g., GENEActiv).

The completeness and accuracy of the dataset is important because periods with missing or erroneous PA tracker data (for instance caused by non-wear time) could result in erroneous behaviour-change detection (false positives) or lack thereof (false negatives). In the event of missing data, U-BEHAVED would skip the detection for that period. We will explain this process further in the next section.

### 4.2. U-BEHAVED Data Pre-Processing

When the discrete time series of steps is streamed to U-BEHAVED, the sum of the steps is stored for that corresponding hour. In case the data is incomplete for any reason, i.e., there is not one full hour worth of data, the sum is not calculated and NULL is stored. The U-BEHAVED data pre-processing produces two vectors, one containing information about the day and hour of the steps recorded, and the other about the total number of steps performed during that day and hour (See Table 1). It could possibly be NULL if data were missing for that hour.

### 4.3. U-BEHAVED Algorithm

**Figure 1:**
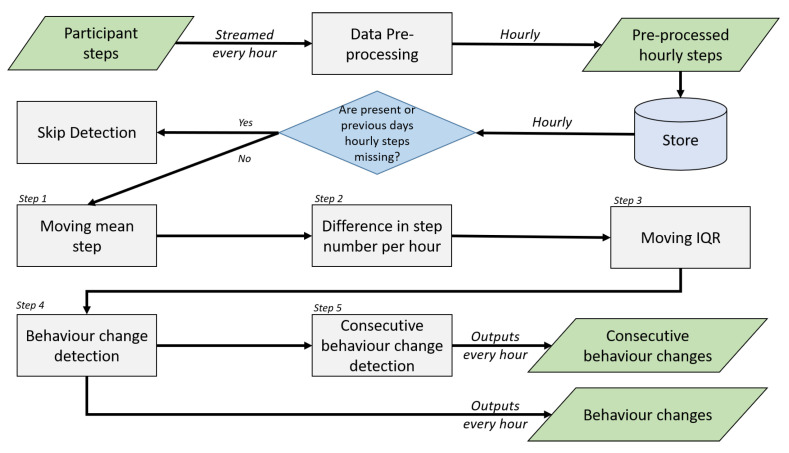
Diagram of U-BEHAVED algorithm steps.

The purpose of the algorithm is to detect significant behaviour changes and new habits as they emerge. Using the two vectors updated during data pre-processing (Section 4.2), it compares current behaviours with recent ones using rolling time windows. The width of the rolling time windows are adjustable to reflect the period used as recent behaviours. The algorithm is executed every hour and is divided into five steps: (i) calculation of the mean number of steps per hour using a rolling time window, (ii) calculation of the step difference per hour, (iii) definition of the upper and lower limits using moving IQR from the step number difference, (iv) classification of step difference outliers as behaviour changes, and (v) classification of consecutive outliers as new habits. If the vector contained NULL (due to missing data, as explained in Section 4.2), the detection for that hour is entirely skipped. The algorithm steps for non-missing data are summarised in Figure 1 and explained below.

**Step 1: Moving mean step number per hour.** The moving mean number of steps per hour is calculated by rolling a time window of *w* days over the hourly steps per day (the two vectors obtained from the pre-processed data) and using Equation (1), where $AvgSteps_{d,h}$ is the mean number of steps per hour at day *d* and hour *h*, and $S_{d,h}$ is the number of steps per hour at day *d* and hour *h*. This results in a vector of the mean step number per hour. If any of $S_{d,h}$ contain a NULL value, the algorithm exits this cycle, skipping the detection.

(1) $$AvgSteps_{d,h}=\sum_{i=d-w}^{d}\frac{S_{i,h}}{w}\;\mathrm{where}\;d>w$$

**Step 2: Difference in step number per hour.** The difference in step number per hour is calculated by subtracting the hourly number of steps per day (vector from the pre-processed data) from the rolling windowed mean number of steps per hour (vector from Step 1) using Equation (2), where DS is the difference in step number per hour for day *d* and hour *h*. This process results in a vector of hourly step differences.

(2) $$DS_{d,h}=S_{d,h}-AvgSteps_{d,h}$$

A positive DS value means that the participant did more steps per hour in the present day compared with the mean number for the *w* days. Conversely, a negative value means that the participant did fewer hourly steps in the present day than in the *w* days.

**Step 3: Moving IQR.** The moving IQR from the last *w* days are calculated by rolling a time window over the difference in step number per hour (vector from Step 2) and using Equation (3), where the IQRs for day *d* are obtained by subtracting from the 75th percentile of the difference in step number per hour (DS) in the last *w* days and the 25th percentile of the difference in step number per hour (DS) in the last *w* days.

(3) $$IQR_{d}=p75_{DS_{d-w},\ldots ,DS_{d-1}}-p25_{DS_{d-w},\ldots ,DS_{d-1}}$$

Then, the upper limit (UL) and lower limit (LL) of day *d* are calculated using the last *w* days (Equations (4) and (5), respectively). This results in two new vectors that contain the daily upper and lower limits.

(4) $$UL_{d}=p75_{DS_{d-w},\ldots DS_{d-1}}+1.5*IQR_{d-1}$$

(5) $$LL_{d}=p25_{DS_{d-w},\ldots ,DS_{d-1}}-1.5*IQR_{d-1}$$

**Step 4: Behaviour-change detection.** As expressed in Equation (6), differences in step number per hour above the daily upper limit (from Step 3) are classified as positive behaviour changes, and differences of steps per hour below the daily lower limit (from Step 3) are classified as negative behaviour changes.

(6) $$Class_{d,h}=\left\{\begin{array}{cc} Positive\;Behaviour\;Change & \mathrm{if}\;DS_{d,h}>UL_{d}\\ Negative\;Behaviour\;Change & \mathrm{if}\;DS_{d,h}<LL_{d}\\ No\;Behaviour\;Change & \mathrm{otherwise} \end{array}\right.$$

**Step 5: Consecutive behaviour-change detection.** For each detected behaviour change, Equation (7) is used to calculate the difference between the hourly step number from the pre-processing data and the mean step number per hour from when the behaviour change was initially detected.

(7) $$DS_{d,h,d_{detection},h_{detection}}=S_{d,h}-AvgSteps_{d_{detection},h_{detection}}$$

Then, this difference is compared with the limits when the behaviour change was initially detected using Equation (8). If it is consecutively higher than the upper limit, the behaviour change is classified as a positive habit. If it is consecutively lower than the lower limit, it is classified as a negative habit.

(8) $$Habit_{d,h,d_{detection},h_{detection}}=\left\{\begin{array}{cc} Yes & \mathrm{if}\;DS_{d,h,d_{detection},h_{detection}}>UL_{d_{detection}}\;\mathrm{and}\;\mathrm{consecutive}\\ No & \mathrm{if}\;DS_{d,h,d_{detection},h_{detection}}<LL_{d_{detection}}\;\mathrm{or}\;\mathrm{non}-\mathrm{consecutive} \end{array}\right.$$

### 4.4. Output

The algorithm output is represented by two data frames. The first data frame (Table 2) contains in each row a detected behaviour change, and in the columns it contains the day and hour when the behaviour changes occurred, the number of additional (or fewer) steps made, and if the behaviour changes were positive or negative. The second data frame (Table 3) contains in each row a detected behaviour change sustained over time, and in each column it contains the day and hour when the sustained behaviour change was first detected, the day when it was last detected, and whether the sustained behaviour changes were positive or negative.

### 4.5. Illustration

**Figure 2:**
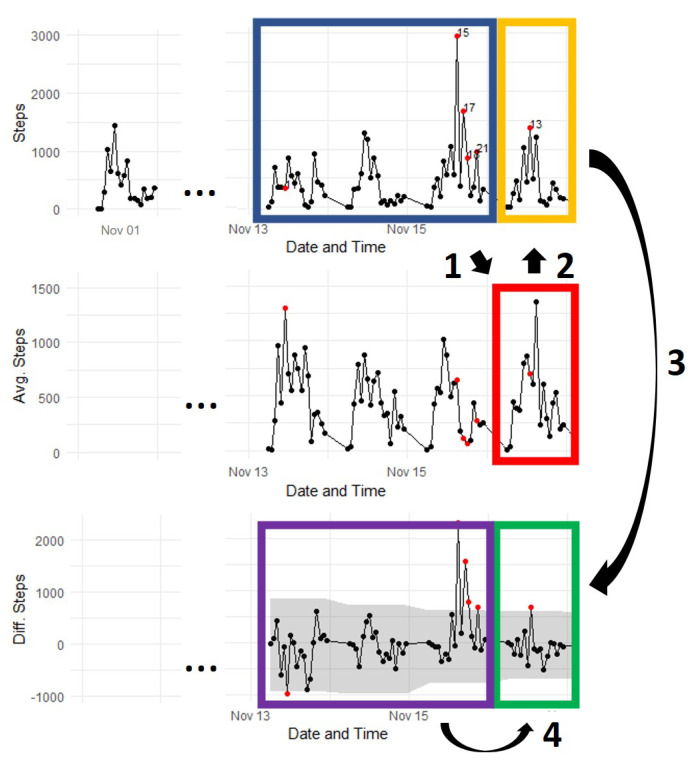
Illustration of first four steps of U-BEHAVED algorithm. Arrows and numbers represent algorithm steps and coloured boxes highlight relevant data of each step. X−axis represents date and time (in hours). Y−axis represents total number of steps (upper graph), mean number of steps using 3-day window (middle graph), and difference between present day and mean number of steps (lower graph). Black dots: data points; red dots: detected behaviour changes; and grey area: IQR.

To illustrate the U-BEHAVED steps, we used data of one participant from a previous health education intervention [29]. We defined the width of the rolling time window as three days (w=3) because the intervention had educational content delivered every three days. Figure 2 shows the first four steps of the U-BEHAVED algorithm. In Step 1, the time window was rolled through the hourly steps (blue box) to calculate the mean step number per hour (red box). In Step 2, the hourly steps were subtracted from the mean number of steps per hour (yellow box) to generate the hourly differences in step number (green box). In Step 3, the IQR limits were calculated (purple box), and in Step 4 they were used as thresholds to detect significant behaviour changes (green box).

**Figure 3:**
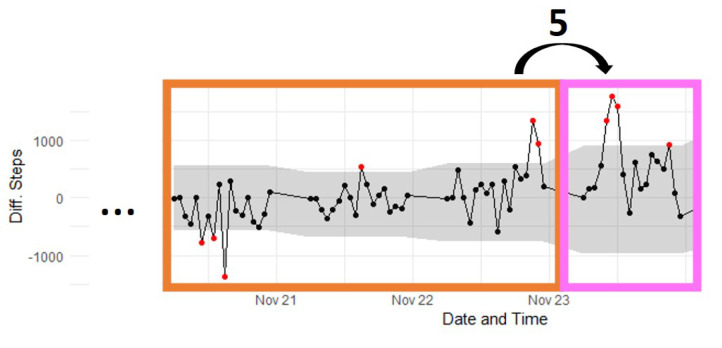
Illustration of Step 5 of U-BEHAVED algorithm. Arrow and number represent Step 5 and coloured boxes highlight relevant processes. X−axis indicates time (in hours). Y−axis is difference in step number.

Step 5 of the U-BEHAVED algorithm is described in Figure 3. It scans the step number difference (pink box) using the IQR of the previously detected behaviour changes (orange box). If the step number difference is outside the IQR limits of previously detected behaviours, the behaviour change is sustained and is labelled as a habit.

## 5. Evaluation

**Figure 4:**
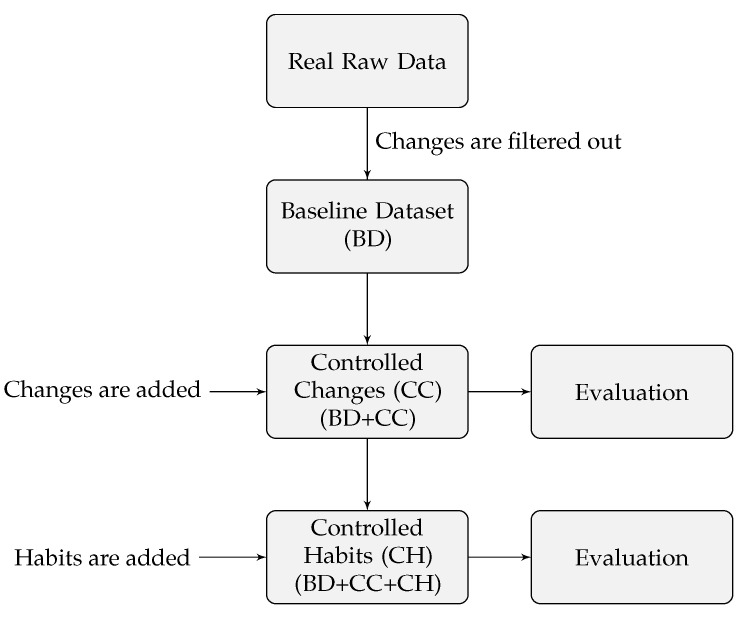
Method to evaluate U-BEHAVED accuracy in detecting behaviour changes and habits.

We used PA tracker data with pre-labelled PA behaviour changes and habits to calculate the accuracy of the U-BEHAVED algorithm. It was constructed from real PA tracker data collected as part of health studies (Real Raw Data) where all significant PA changes were smoothed out to remove all behaviour changes and habits (Baseline Dataset). We set the width of the algorithm rolling window to a short period (w=3 days) and added different magnitudes of PA behaviours (Baseline Dataset + Controlled Changes) and habits (Baseline Dataset + Controlled Changes + Controlled Habits) in a controlled manner. We simulated an hourly stream of data to the algorithm, and for each added magnitude of PA behaviour, we calculated the algorithm accuracy as the ratio of detections relative to all added behaviours. The evaluation method is summarised in Figure 4.

### 5.1. Construction of the Evaluation Dataset

The real raw data contained records of 79 participants who used a PA tracker that continuously recorded their number of steps per minute. We divided the 79 participants into two groups. The first group included 30 children who participated in a school-based health education programme [29,30] (median number of hourly steps = 320, mean hourly step coefficient of variation = 50%), and each wore a Misfit wearable sensor [31]. The second group included 49 adults from two public datasets [32,33] (median number of hourly steps = 218, mean hourly step coefficient of variation = 140%), and each wore a Fitbit wearable sensor [34].

Using the Real Raw Data, we built the Baseline Dataset (BD) free of behaviour changes and habits. We first pre-processed the data for each participant (Section 4.2) and calculated the mean step number per hour for each participant. Then, we replicated this nine times for each participant, simulating nine equal days of PA. We needed nine days of PA because we used the first six days as baseline (the width of the algorithm rolling window was set to three days), and then we added behaviour changes in the last three days to evaluate the algorithm. We simulated the participants’ natural PA variability [25,26] by introducing (or removing) steps per hour based on each participant’s hourly step coefficient of variation, calculated from the pre-processed Real Raw Data. We manually inspected the resulting dataset of 12,798 data points (79 participants × 18 h × 9 days) for sharp increases or decreases in the number of steps per hour to confirm that behaviour changes were not involuntarily introduced.

### 5.2. Evaluation of Behaviour-Change Detection

We incorporated two behaviour changes to the BD of each participant on day seven: one positive behaviour change at 8 am by adding PA (steps), and one negative behaviour change at noon by removing PA (steps) (Figure 4). We chose these specific times because they are times when participants may generate behaviour changes, such as walking to school/workplace in the morning and using a computer or smartphone at lunchtime. We also varied the number of steps.

**Figure 5:**
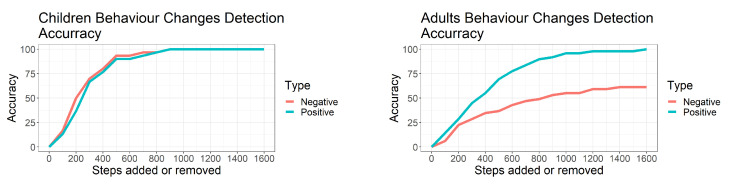
Left and right graph show behaviour-change detection accuracy of children and adults, respectively. X−axis indicates number of steps added at 8 am as positive behaviour changes or removed at noon as negative behaviour changes. Y−axis indicates percentage of behaviour changes detected relative to all changes added. Red and blue lines indicate percentage of negative and positive behaviour changes detected, respectively.

Figure 5 shows the detection accuracy for various amounts of steps. As expected, as more steps were added or removed to simulate behaviour changes, the accuracy of the algorithm increased sharply.

For the children’s dataset, the algorithm detected ∼80% of the positive and negative behaviour changes when the added changes corresponded to at least 400 steps per hour. This detection accuracy reached 100% when the added changes corresponded to at least 900 steps per hour.

For the adults’ dataset, the algorithm detected ∼80% and 100% of positive behaviour changes when the added changes corresponded to at least 600 steps and 1600 steps per hour, respectively. The detection of negative behaviour changes by the algorithm reached 61% when the added changes corresponded to at least 1400 steps per hour. Overall, 80% of all (positive and negative) added behaviour changes were detected at 1600 steps.

### 5.3. Habit Detection Evaluation

Similar to the behaviour-change detection evaluation, we incorporated two habits for each participant from day 7 to day 9: one positive habit at 8 am by adding the same amount of PA (steps) during the three consecutive days, and one negative habit at noon by removing the same amount of PA (steps) during the three consecutive days (Figure 4). The habit was labelled as detected only if it was consecutively detected from day 7 to day 9.

**Figure 6:**
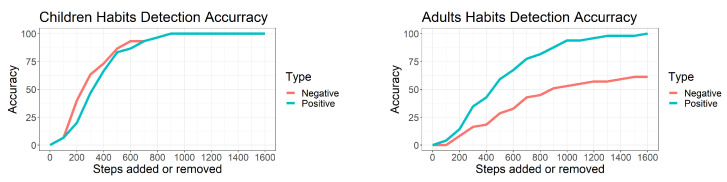
Left and right graphs show habit detection accuracy for children and adults, respectively. X−axis indicates number of steps added at 8 am (positive behaviour changes) or removed at noon (negative behaviour changes). Y−axis indicates percentage of habits detected in function of number of steps added. Red and blue lines indicate percentage of negative and positive habits detected, respectively.

Figure 6 shows the detection accuracy at various amount of steps. Before the addition of the two habits (i.e., addition = 0), the algorithm did not detect any habit, as expected. Then, the number of detected habits increased sharply as these became more pronounced.

For the children’s dataset, the algorithm detected ∼80% of the positive and negative habits when the added habits corresponded to at least 500 steps per hour. This detection accuracy reached 100% when the added habits corresponded to at least 900 steps per hour.

For the adults’ dataset, the algorithm detected ∼80% of positive habits when at least 700 steps per hour were added and increased to 100% following addition of at least 1600 steps per hour. The detection of negative habits by the algorithm reached 61% upon addition of at least 1500 steps per hour. Overall, 80% of all (positive and negative) added habits were detected at 1600 steps.

## 6. Discussion and Conclusions

This article presents U-BEHAVED, an unsupervised algorithm to detect PA behaviour changes and new habits as they appear. The algorithm identifies significant changes in current behaviours by comparing them to recent past behaviours using rolling time windows of participants’ step count data captured in real time using wearable PA trackers. The detection of PA behaviour changes and new habits as they appear represents valuable information for a PA promotion strategy because it can help to increase its effectiveness and the participants’ adherence by enabling personalisation [35]. For instance, by detecting and understanding how participants change their behaviours in real time, PA promotion strategies can be adjusted to each participant’s needs, and relevant personalised feedback can be generated. It can also help to assess and better understand how the programme or intervention influences participants by analysing their behaviour changes and new habits after the PA promotion strategy ends. It may also reveal how the participants’ behaviour changes affect other lifestyle behaviours, such as sleep and diet.

The evaluation of U-BEHAVED detection accuracy using data from 79 users showed that it can successfully detect significant PA behaviour changes and new habits, even when subtle, in the general population with a non-pathological gait. In the children’s dataset (lower PA variability), the algorithm detected 100% of behaviour changes and new habits when a difference of at least 900 steps per hour was added. In the adult dataset (higher PA variability), U-BEHAVED detected 80% of behaviour changes when a difference of at least 1600 steps per hour was added. The difference in the number of steps needed to detect behaviour changes and new habits was lower in the children’s than in the adults’ dataset because the change detection by U-BEHAVED is based on the participants’ PA variability, which was lower in the children’s than adults’ dataset. The algorithm can detect behaviour changes and habits as subtle as a 100 steps difference per hour if they are significant, for instance for a sedentary participant with low PA variability. The difference in steps would approximate 9 and 16 min of physical activity at moderate-to-vigorous intensity for the children and adult datasets, respectively, based on a threshold cadence of around 100 steps per minute, which represents a habitual walking pace [36,37,38]. Examples of behaviour changes and habits that can be detected in children are walking with their parents to school in the morning [39,40] and playing an active game at lunchtime [41], and for adults, examples include active commuting [42] and exercising at lunchtime [43].

The U-BEHAVED algorithm can be easily implemented in the framework of any strategy that promotes PA or teaches healthy behaviours and relies on PA trackers because it uses as input data the number of steps performed by participants (i.e., the output data of most commercial, smartphone, and medical-grade PA trackers). The algorithm can also be easily adapted to detect behaviour changes at any programme content delivery pace because the algorithm’s rolling window can be set to any length. For instance, if the health intervention delivery pace is weekly, the rolling window can be set to 7 days. The flexibility to adjust the window length enables comparisons of past behaviour in the short, medium, and long term. For instance, to detect changes in current behaviour compared with the previous month, the rolling window can be set to 30 days.

U-BEHAVED uses a step aggregation level per hour to detect PA behaviour changes as they occur. This aggregation level was selected because it is coarse enough to detect intra-day changes and avoid mislabelling any insignificant variation in the step number as a change. Other aggregation levels could be used to detect other behaviour-change types; however, this would lead to new misclassification issues. For instance, a lower aggregation level, such as per minute, might allow for identifying more subtle behavioural changes but it might mislabel small PA bursts as behaviour changes when they are not. Additional methods need to be developed for each aggregation level to avoid misclassification.

We note that while steps captured by physical activity trackers are acknowledged as being a reliable high-level indicator of a person’s total amount of physical activity [44] with a high step activity recognition rate [45], they may not capture all physical activities. U-BEHAVED focuses on detecting behaviour changes in physical activity that can be defined as steps and excludes other non-ambulatory activities, such as cycling [46]. Future work can explore the inclusion of user-defined activities into the algorithm, as well as the inclusion of additional data that may help in detecting the presence of a non-ambulatory activity, such as intensity levels. This would also address the issue of missing PA data caused by the removal of the tracker (non-wear time) or by a technical error during recording or synchronisation.

Furthermore, the detection of other types of habits can be explored. We flagged as new habits behaviour changes that were performed consecutively every day at the same hour; however, different behaviour changes can be performed at different times of the day, non-consecutively or with a broader difference of time, such as doing sport only on Mondays. This suggests that many types of habits remain to be detected.

## Figures and Tables

**Table 1:** Example of total number of steps per hour.

Day Hour	Total Number of Steps
1 November 2021 08:00	390
1 November 2021 09:00	564
1 November 2021 10:00	1046
…	…

**Table 2:** Example of data frame with detected behaviour changes.

Day and Hour	Step Number Difference	Type of Behaviour Change Detected
11 November 2021 11:00	−956	Negative
15 November 2021 15:00	2300	Positive
15 November 2021 17:00	1549	Positive
…	…	

**Table 3:** Example of data frame with detected sustained behaviour changes.

First Day and Hour	Last Day	Type of Habit
16 November 2021 13:00	18 November 2021	Positive
18 November 2021 14:00	19 November 2021	Positive
21 November 2021 08:00	25 November 2021	Negative
…	…	

## Data Availability

Not applicable.

## References

[1] Yang C.C., Hsu Y.L. (2010). A Review of Accelerometry-Based Wearable Motion Detectors for Physical Activity Monitoring. Sensors.

[2] Santos O.C. (2015). Education still Needs Artificial Intelligence to Support Personalized Motor Skill Learning: Aikido as a Case Study. http://ceur-ws.org/Vol-1432/ai_ed_pap9.pdf.

[3] Dias Pereira dos Santos A., Yacef K., Martinez-Maldonado R. (2017). Forró Trainer: Automated Feedback for Partner Dance Learning. Proceedings of the Adjunct Publication of the 25th Conference on User Modeling, Adaptation and Personalization, UMAP ’17.

[4] Martinez-Maldonado R., Yacef K., Santos A.D.P.D., Shum S.B., Echeverria V., Santos O.C., Pechenizkiy M. Towards Proximity Tracking and Sensemaking for Supporting Teamwork and Learning. Proceedings of the IEEE 17th International Conference on Advanced Learning Technologies, ICALT 2017.

[5] Galy O., Yacef K., Caillaud C. (2019). Improving Pacific Adolescents’ Physical Activity Toward International Recommendations: Exploratory Study of a Digital Education App Coupled With Activity Trackers. JMIR mHealth uHealth.

[6] Lee P.H., Yu Y.Y., McDowell I., Leung G.M., Lam T.H. (2013). A cluster analysis of patterns of objectively measured physical activity in Hong Kong. Public Health Nutr..

[7] Díaz C., Galy O., Caillaud C., Yacef K. (2020). A Clustering Approach for Modeling and Analyzing Changes in Physical Activity Behaviors From Accelerometers. IEEE Access.

[8] Fukuoka Y., Zhou M., Vittinghoff E., Haskell W., Goldberg K., Aswani A. (2018). Objectively measured baseline physical activity patterns in women in the mped trial: Cluster analysis. J. Med. Internet Res..

[9] Mollee J.S., Klein M.C.A., Benferhat S., Tabia K., Ali M. (2017). Empirical Validation of a Computational Model of Influences on Physical Activity Behavior. Advances in Artificial Intelligence: From Theory to Practice.

[10] Sprint G., Cook D.J., Schmitter-Edgecombe M., Holmes D.E., Jain L.C. (2016). Unsupervised detection and analysis of changes in everyday physical activity data. Journal of Biomedical Informatics.

[11] Chousiadas D., Menychtas A., Tsanakas P., Maglogiannis I. (2018). Advancing Quantified-Self Applications Utilizing Visual Data Analytics and the Internet of Things. Proceedings of the IFIP International Conference on Artificial Intelligence Applications and Innovations.

[12] Aguilera A., Figueroa C.A., Hernandez-Ramos R., Sarkar U., Cemballi A., Gomez-Pathak L., Miramontes J., Yom-Tov E., Chakraborty B., Yan X. (2020). mHealth app using machine learning to increase physical activity in diabetes and depression: Clinical trial protocol for the DIAMANTE Study. BMJ Open.

[13] Angelides M.C., Wilson L.A.C., Echeverría P.L.B. (2018). Wearable data analysis, visualisation and recommendations on the go using android middleware. Multimed. Tools Appl..

[14] Chen H.K., Chen F.H., Lin S.F. (2021). An ai-based exercise prescription recommendation system. Appl. Sci..

[15] Forman E.M., Kerrigan S.G., Butryn M.L., Juarascio A.S., Manasse S.M., Ontañón S., Dallal D.H., Crochiere R.J., Moskow D. (2019). Can the artificial intelligence technique of reinforcement learning use continuously-monitored digital data to optimize treatment for weight loss?. J. Behav. Med..

[16] Mollee J.S., Araújo E.F.M., Manzoor A., van Halteren A.T., Klein M.C.A. (2017). Explaining Changes in Physical Activity Through a Computational Model of Social Contagion. Springer Proceedings in Complexity.

[17] Rabbi M., Pfammatter A., Zhang M., Spring B., Choudhury T. (2015). Automated Personalized Feedback for Physical Activity and Dietary Behavior Change With Mobile Phones: A Randomized Controlled Trial on Adults. JMIR mHealth uHealth.

[18] Zhou Z., Dai W., Eggert J., Giger J.T., Keller J., Rantz M., He Z. A real-time system for in-home activity monitoring of elders. Proceedings of the 31st Annual International Conference of the IEEE Engineering in Medicine and Biology Society: Engineering the Future of Biomedicine (EMBC 2009).

[19] Zhu J., Dallal D.H., Gray R.C., Villareale J., Ontañón S., Forman E.M., Arigo D. (2021). Personalization Paradox in Behavior Change Apps. Proc. ACM Hum.-Comput. Interact..

[20] Gasparetti F., Aiello L.M., Quercia D. (2020). Personalized weight loss strategies by mining activity tracker data. User Model. User-Adapt. Interact..

[21] Batool T., Vanrompay Y., Neven A., Janssens D., Wets G. (2019). CTASS: An intelligent framework for personalized travel behaviour advice to cardiac patients. J. Ambient. Intell. Humaniz. Comput..

[22] Schäfer H., Bachner J., Pretscher S., Groh G., Demetriou Y. Study on Motivating Physical Activity in Children with Personalized Gamified Feedback. Proceedings of the Adjunct Publication of the 26th Conference on User Modeling, Adaptation and Personalization (UMAP ’18), Association for Computing Machinery.

[23] Dijkhuis T.B., Blaauw F.J., van Ittersum M.W., Velthuijsen H., Aiello M. (2018). Personalized physical activity coaching: A machine learning approach. Sensors.

[24] Hermens H., op den Akker H., Tabak M., Wijsman J., Vollenbroek M. (2014). Personalized Coaching Systems to support healthy behavior in people with chronic conditions. J. Electromyogr. Kinesiol. Off. J. Int. Soc. Electrophysiol. Kinesiol..

[25] Baranowski T., de Moor C. (2000). How many days was that? Intra-individual variability and physical activity assessment. Res. Q. Exerc. Sport.

[26] Jaeschke L., Steinbrecher A., Jeran S., Konigorski S., Pischon T. (2018). Variability and reliability study of overall physical activity and activity intensity levels using 24 h-accelerometry-assessed data. BMC Public Health.

[27] Ben-Gal I. (2005). Outlier detection. Data Mining and Knowledge Discovery Handbook.

[28] Kokoska S., Zwillinger D. (1999). CRC Standard Probability and Statistics Tables and Formulae, Student Edition.

[29] Yacef K., Caillaud C., Galy O. (2018). Supporting Learning Activities with Wearable Devices to Develop Life-Long Skills in a Health Education App. Artificial Intelligence in Education.

[30] Caillaud C., Ledger S., Diaz C., Clerc G., Galy O., Yacef K. (2022). iEngage: A digital health education program designed to enhance physical activity in young adolescents. PLoS ONE.

[31] Misfit Fossil Group. https://www.misfit.com/.

[32] Brinton J.E., Keating M.D., Ortiz A.M., Evenson K.R., Furberg R.D. (2017). Establishing linkages between distributed survey responses and consumer wearable device datasets: A pilot protocol. JMIR Res. Protoc..

[33] Thambawita V., Hicks S.A., Borgli H., Stensland H.K., Jha D., Svensen M.K., Pettersen S.A., Johansen D., Johansen H.D., Pettersen S.D. (2020). PMData: A Sports Logging Dataset. Proceedings of the 11th ACM Multimedia Systems Conference, MMSys ’20.

[34] Fitbit Google. https://www.fitbit.com/.

[35] Lustria M.L.A., Noar S.M., Cortese J., Van Stee S.K., Glueckauf R.L., Lee J. (2013). A meta-analysis of web-delivered tailored health behavior change interventions. J. Health Commun..

[36] Murtagh E.M., Mair J.L., Aguiar E., Tudor-Locke C., Murphy M.H. (2021). Outdoor Walking Speeds of Apparently Healthy Adults: A Systematic Review and Meta-analysis. Sport. Med..

[37] Olds T.S., Ridley K., Dollman J., Maher C.A. (2010). The validity of a computerized use of time recall, the multimedia activity recall for children and adolescents. Pediatr. Exerc. Sci..

[38] Tudor-Locke C., Craig C.L., Brown W.J., Clemes S.A., De Cocker K., Giles-Corti B., Hatano Y., Inoue S., Matsudo S.M., Mutrie N. (2011). How many steps/day are enough? For adults. Int. J. Behav. Nutr. Phys. Act..

[39] Chillón P., Panter J., Corder K., Jones A., Van Sluijs E. (2015). A longitudinal study of the distance that young people walk to school. Health Place.

[40] Merom D., Tudor- Locke C., Bauman A., Rissel C. (2006). Active commuting to school among NSW primary school children: Implications for public health. Health Place.

[41] Ridley K., Zabeen S., Lunnay B.K. (2018). Children’s physical activity levels during organised sports practices. J. Sci. Med. Sport.

[42] Shephard R.J. (2008). Is active commuting the answer to population health?. Sport. Med..

[43] Gjestvang C., Stensrud T., Hansen B.H., Kolle E., Haakstad L.A. (2020). Are fitness club members likely to meet the current physical activity recommendations?. Transl. Sport. Med..

[44] Tudor-Locke C., Bassett D.R. (2004). How many steps/day are enough?. Sport. Med..

[45] Attal F., Mohammed S., Dedabrishvili M., Chamroukhi F., Oukhellou L., Amirat Y. (2015). Physical Human Activity Recognition Using Wearable Sensors. Sensors.

[46] Miller R., Brown W., Tudor-Locke C. (2006). But what about swimming and cycling? How to “count” non-ambulatory activity when using pedometers to assess physical activity. J. Phys. Act. Health.

